# Revamping of a Full-Scale Membrane Plant for Landfill Leachate Pretreatment Using Partial Nitritation

**DOI:** 10.3390/membranes14050115

**Published:** 2024-05-14

**Authors:** Laura Palli, Francesca Tuci, Letizia Macellaro La Franca, Donatella Fibbi, Riccardo Gori

**Affiliations:** 1 Gestione Impianti Depurazione Acque Spa, Via di Baciacavallo 36, 59100 Prato, Italy; 2 Department of Civil and Environmental Engineering, University of Florence, Via S. Marta 3, 50139 Florence, Italy

**Keywords:** landfill leachates, partial nitritation, MBR, energy savings

## Abstract

This paper describes a case study involving a revamping of a full-scale membrane bioreactor that treats landfill leachate and other liquid wastes. The main change was the introduction of nitritation/denitritation in alternating cycles instead of the classic denitrification/nitrification process, together with the installation of fine bubble diffusers, a reduction in the volume of the biological compartment, and an increase in the equalization volume. The most significant results were obtained for the biological compartment, with a decrease in the specific energy consumption of 46.6%. At the same time, the removal efficiency of COD, BOD, and TN substantially remained the same before and after plant revamping, while the removal efficiency of TP increased over the years, reaching an average value of almost 71%. Regarding the ultrafiltration unit, the specific flux (or permeability) was characterized by an increasing trend. At the same time, the specific energy consumption of this section decreased by 9.4%. These results led to the conclusion that the changes introduced with the revamp led to a more stable process, a reduction in membrane fouling, and important energy savings.

## 1. Introduction

Landfill leachate is a type of wastewater characterized by high ammonium content and a high amount of non-biodegradable compounds [1]. To adequately remove nitrogen, especially in mature landfill leachate, biological treatments are preferred, and a biological removal of nitrogen is generally achieved via the conventional nitrification–denitrification process [2]. This type of approach has been considered a good choice because it is economical, effective, and easy to operate [3]. However, if the initial objective in wastewater treatment was primarily focused on enhanced nutrients removal to meet the more stringent effluent requirements, current goals aim to maximize energy and resource recovery [2]. In this context, many researchers have developed and tested a partial nitrification process for more efficient N removal in wastewater treatment systems [2,4,5]. Partial nitrification or nitritition includes conversion of ammonium (NH_4_^+^) only to nitrite (NO_2_^−^) by ammonium oxidizing bacteria (AOB) without further oxidation to nitrate (NO_3_^−^), followed by further degradation of nitrite into N_2_ gas under anoxic conditions (denitritation). Compared to conventional nitrification–denitrification, partial nitrification via nitrite presents four significant advantages: (a) requires 25% less energy in the aerobic phase [5] because while complete nitrification requires 4.57 mgO_2_/mgN, nitritation requires only 3.43 mgO_2_/mgN; (b) reduces the COD requirements in the anoxic phase up to 40%, which can be extremely useful for the treatment of wastewater with low C/N ratio; (c) reduces biomass production [3,4]; and (d) reduces carbon dioxide emission during the denitrification phase by 20% [3,4].

The key to achieving partial nitrification lies in nitrite accumulation achieved by the accumulation of ammonia oxidizing bacteria (AOB) and the inhibition or washout of the nitrite-oxidizing bacteria (NOB) in reactors [6]. Both AOB and NOB are sensitive to parameters such as dissolved oxygen (DO), temperature, pH, free ammonia (FA), and free nitrous acids (FNAs). Based on these parameters, several effective control strategies can be adopted to inhibit NOB growth and achieve successful partial nitrification [7].

In the literature, low DO concentrations (lower than 1 mg/L) have been successfully applied to achieve partial nitrification for landfill leachate [6], exploiting the difference in the oxygen affinity constant of AOB and NOB (KOA = 0.74 ± 0.02 mg/L and KON = 1.75 ± 0.01 mg/L) [8].

In this context, this paper describes a full-scale MBR treating liquid wastes (mainly composed by landfill leachate) by comparing the energy demand before and after a revamping that introduced the partial nitrification technology. Furthermore, the effect of the different biological denitrification pathways on membrane fouling is investigated.

## 2. Materials and Methods

### 2.1. Plant Description

**Figure 1:**
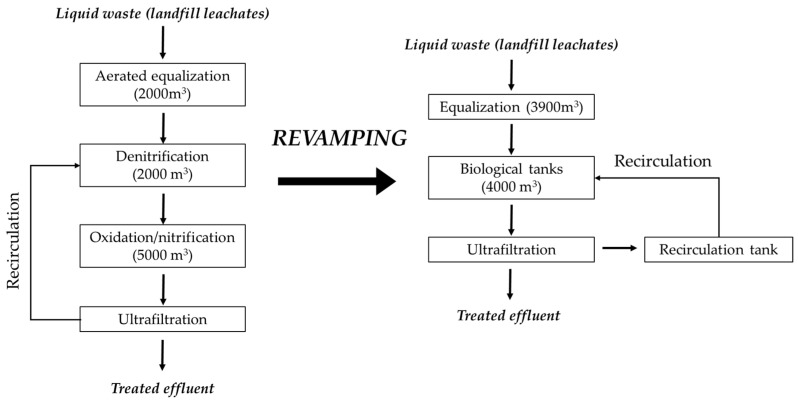
Simplified flow diagram of the side-stream plant before and after the revamping.

The MBR plant is part of a side-stream wastewater treatment plant (WWTP) designed to pre-treat a mixture of landfill leachates prior to being discharged in the main line of a full-scale WWTP that treats municipal and industrial wastewater (Calice WWTP in Prato, managed by G.I.D.A. SpA, Prato, Italy). The main aim of the side-stream plant is to intensively treat the mixture of leachates in a separate plant, since they represent less than 2% of the total volume treated by the main WWTP but possess more than 50% of the total COD entering the plant. The old plant has been described elsewhere [9], and the treatment train (Figure 1) consisted of the following sections: (a) an aerated equalization tank, with a volume of 2000 m^3^, where all the leachates are discharged and mixed together; (b) a denitrification tank, with a volume of 2000 m^3^, equipped with a dissolved oxygen (DO) probe, a pH probe, and a redox probe; (c) an oxidation/nitrification tank, with a volume of 5000 m^3^, equipped with 6 rotor brushes as a surface aeration system, a submerged mixing system, and probes for the measurement of DO, pH, and redox potential; and (d) two ultrafiltration units placed in an external tank. After the revamping (Figure 1), the treatment train is composed of (a) six equalizations tanks, with a total volume of approximately 3900 m^3^, where the leachate and the other wastes are discharged and mixed in the desired proportions; (b) two biological tanks, each a volume of 2000 m^3^, equipped with one pH probe, two ORP probes, two DO probes, and one TSSs probe; and (c) two ultrafiltration units, not changed after the revamping. Moreover, the aeration system was modified and fine bubble diffusers were installed in the biological tanks, together with volumetric blowers and submerged propeller mixers for the anoxic phase.

### 2.2. Biological Compartment

The biological compartment is the single process that changed the most during the revamping, with the introduction of nitritation/denitritation in alternating cycles instead of the classic denitrification/nitrification process. To this end, the main changes were (a) the use of a smaller tank, 4000 m^3^ total, instead of 7000 m^3^ (as a sum of denitrification and nitrification tanks); (b) the installation of submerged mixers to be used during the anoxic phase; (c) the installation of porous membrane diffusers; (d) the installation of volumetric blowers to supply air to the biological process; (e) the installation of DO, ORP, pH, and TSSs probes for process control and monitoring; and (f) the installation of a dedicated software for control of the nitritation–denitritation process. The system is automatically managed by a patented automatic control device [10]. The control system made it possible to determine the optimal duration of the aerobic and anoxic phases by analysing dissolved oxygen and oxidation-reduction potential data. The DO varies from 0.2 to 1.0 mg/L during the aerated phase and the ORP generally varies between −100 mV and +200 mV.

### 2.3. Ultrafiltration

The membrane filtration module of the side-stream plant is placed into two filtration tanks with a volume of 40 m^3^ each, divided into two independent sections, each containing a train of membranes for a total of 4 trains, 36 modules, and a total filtration surface of 2274 m^2^. The chosen membranes were Zenon^®^ (Oakville, Ontario, now Suez^®^) polyvinylidene fluoride (PVDF) hollow fibre, with a porosity of 0.04 µm, which were completely submerged in the mixed liquor, with an “outside-inside” filtration system, meaning that the permeate was collected in the lumen by vacuum. The air scouring was performed using Leap^®^ technology, which generated large bubbles that removed more debris per volume of air. The average airflow for membrane scouring was 110 Nm^3^/h. Membranes were generally operated at constant flux and variable TMP, setting the needed flow for the waste to be treated and registering the resulting TMP.

The trains have a filtration cycle of 1185 s, composed as follows: filtration (360 s), relaxation (30 s), filtration (360 s), relaxation (30 s), filtration (360 s), backwash (45 s). The membranes were cleaned once a week with 200 mg/L of sodium hypochlorite for 360 s and once a week with 2000 mg/L of citric acid for 360 s. During this operation, which was performed automatically, chemicals were added to the permeate for the backwashing without having to empty the tank or remove the membranes. Once a year, a recovery cleaning was performed with 1100 mg/L of sodium hypochlorite and 2200 mg/L of citric acid. In this case, the filtration tank was emptied, filled with water and chemicals, and then the membranes were left to soak for at least 8 h.

## 3. Results

### 3.1. Characteristics of the Influent Mixture

The main objective of implementing alternating cycles in the biological compartment of the old plant was to achieve savings in terms of energy consumption without compromising the performances of the treatment. The evaluation of the obtained results was carried out by comparing two well-defined time periods: the three-year period of 2015–2017, before the revamping, and the three-year period of 2021–2023, after the revamping. Table 1 reports the characteristics of the influent wastewater referring to the two three-year periods, respectively. The values are expressed as average, minimum, and maximum.

Comparing the data in Table 1, before and after the revamping, an increase in the average concentration in the influent mixture can be seen for TSS and N-NO_3_, while all the other parameters show a lower average concentration.

Table 2 shows the most relevant operating parameters of the biological compartment in terms of mixed liquor suspended solids (MLSSs), waste flow rate, and sludge retention time (SRT) for the three years before and after the revamping.

Comparing the data in Table 2, referred to the three-year period before and after the revamping, respectively, it can be observed that the MLSSs remained almost constant while the waste flow rate decreased from 415 to 188 (TSS/year). Considering the reduction in the total volume of the biological compartment, the SRT slightly decreased from 311 d to 254 d on average.

### 3.2. Removal Efficiency

**Figure 2:**
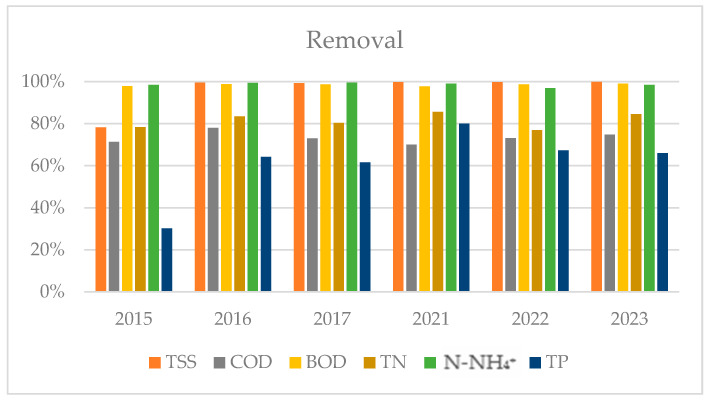
Average annual removal efficiency of TSSs, COD, BOD, total nitrogen (TN), ammonia nitrogen (N-NH_4_^+^), and total phosphorus (TP).

**Figure 3:**
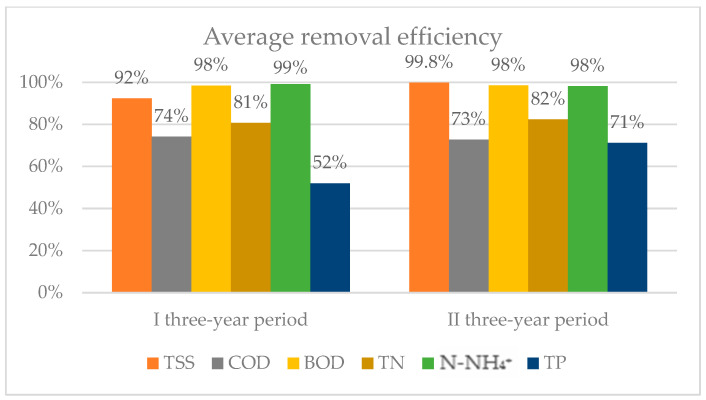
Average removal efficiency in pre- and post-revamping three-year periods.

**Figure 4:**
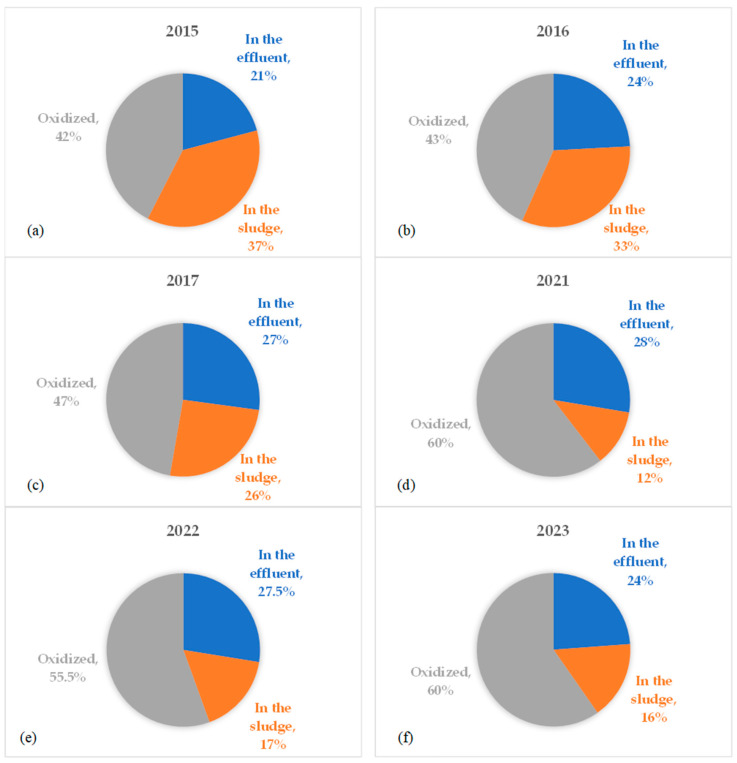
COD mass balance calculation in 2015 (**a**), 2016 (**b**), 2017 (**c**), 2021 (**d**), 2022 (**e**), and 2023 (**f**). The percentages represent the final fate of COD entering the plant.

The annual average removal efficiency of the side-stream plant of total suspended solids (TSSs), COD, BOD_5_, total nitrogen (TN), N-NH_4_^+^, and total phosphorus (TP) is shown in Figure 2. It can be observed that TSSs removal was consistently above 99%, except in 2015. BOD has been constantly removed since 2015, with an average efficiency above 98%. COD removal was variable over the entire analyzed period, with an average removal efficiency of 74% and 72% in the first and second three-year periods, respectively (Figure 3). A COD balance was calculated to better understand its biological removal, considering the annual average influent and effluent concentrations (Figure 4). Regarding nutrient removal, ammonium was removed with an average efficiency higher than 98%, presenting a slight inflection in 2022. TN removal was not constant over time, with an average removal above 80% in both reference periods. Finally, the results show an increase in TP removal after the plant revamping, with an average efficiency that went from 52% (in the period of 2015–2016–2017) to 71% in the period of 2021–2022–2023.

### 3.3. Energy Consumptions

**Figure 5:**
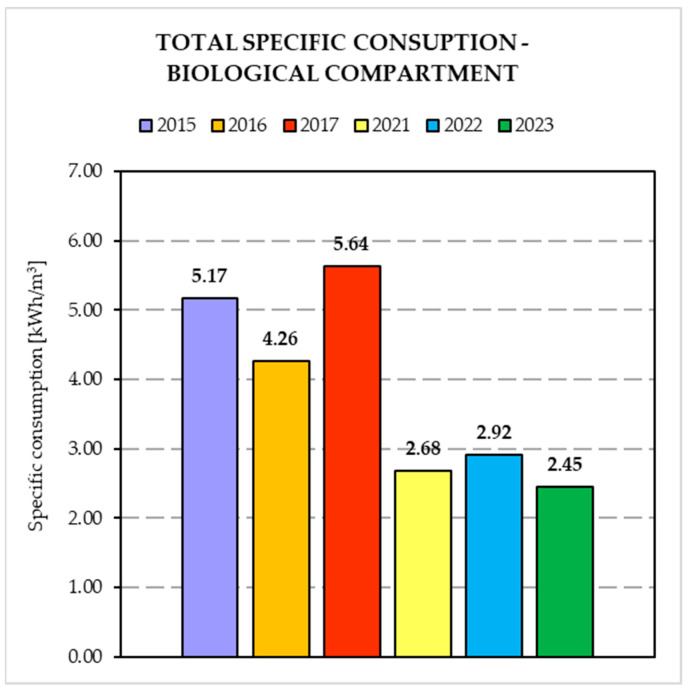
Total specific consumption in biological compartment before and after plant revamping.

**Figure 6:**
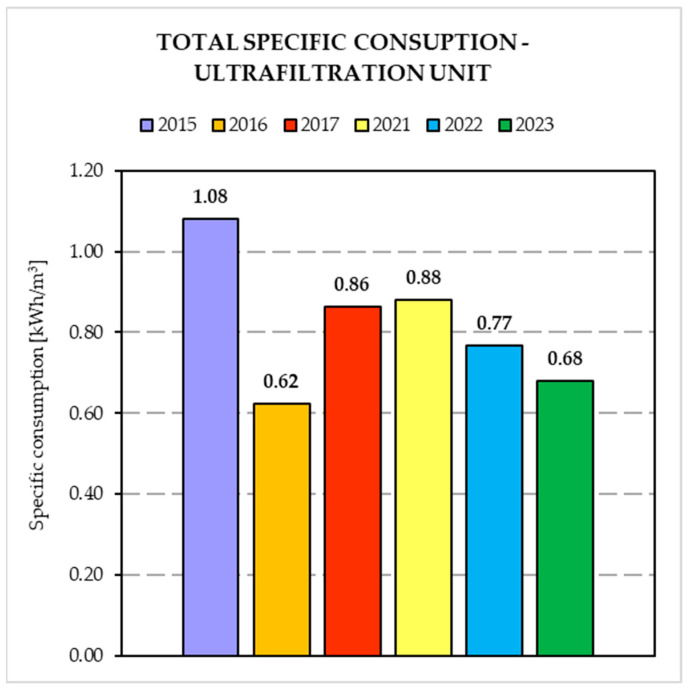
Total specific consumption in Ultrafiltration unit before and after plant revamping.

Figure 5 and Figure 6 show the results obtained from the comparison of the specific energy consumptions relating to the two periods considered. In particular, energy consumption for each treatment unit (i.e., biological compartment and ultrafiltration unit) were considered.

In particular, Figure 5 shows that the most significant results were obtained for the biological compartment with a decrease in the average specific consumption of 46.6%, while in regard to the UF unit, despite the relative specific energy consumption being expected almost constant, a consumption decrease of 9.4% was recorded. As a result, the total specific energy consumption was reduced by 41.2%.

### 3.4. Membrane Treatment

**Figure 7:**
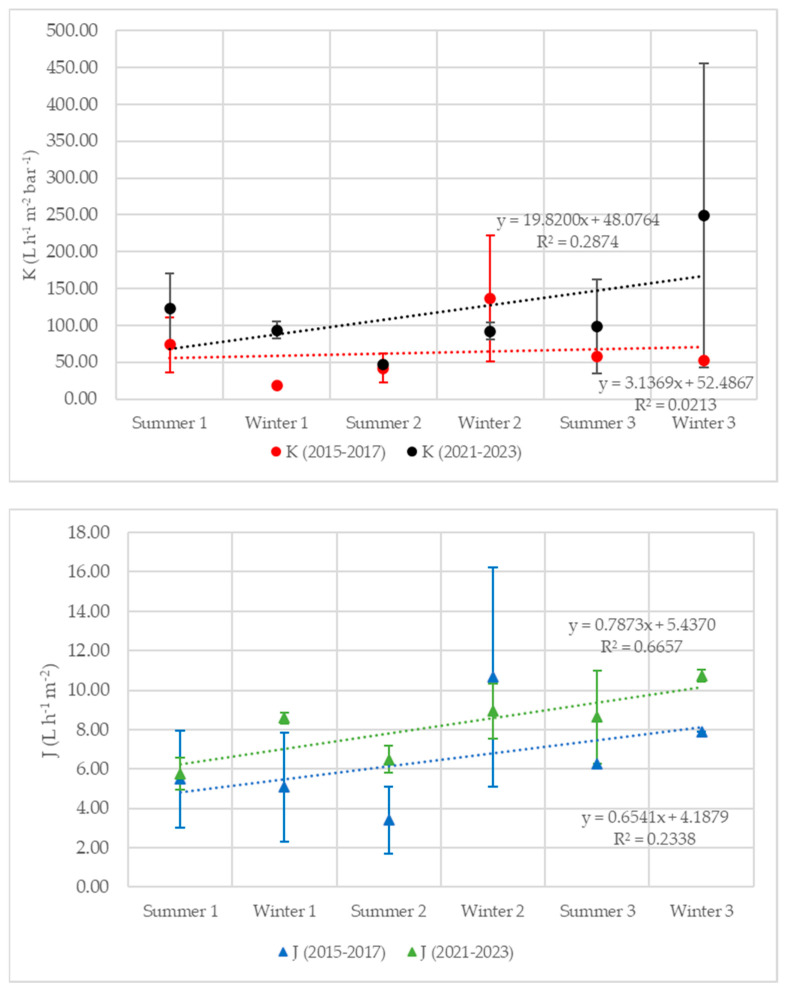
Permeability (K) and permeate flow (J) trends before (2015–2016–2017) and after (2021–2022–2023) plant revamping.

In terms of membrane performance, the flux and the permeability were calculated twice per year, in summer and winter. The results are reported in Figure 7. It can be observed that the flux fluctuates between 3 and 11 L·m^−2^·h^−1^ (6.5 ± 3 L·m^−2^·h^−1^) in the first period, while it increases after the revamping, varying in the range of 6–11 L·m^−2^·h^−1^ (8.2 ± 2 L·m^−2^·h^−1^). Moreover, the flux is generally higher in winter, regardless of the analyzed year. At the same time, the permeability varies between 18 and 137 L·m^−2^·h^−1^·bar^−1^ (63 ± 40 L·m^−2^·h^−1^·bar^−1^) in the first period and between 48 and 123 L·m^−2^·h^−1^·bar^−1^ (117 ± 69 L·m^−2^·h^−1^·bar^−1^) in the second one, showing an increasing trend.

## 4. Discussion

### 4.1. Biological Section

The average TSSs removal is always above 99%, in line with the typical TSSs rejection of ultrafiltration membranes [11], except for in 2015. As already highlighted by [9], during this year, the influent TSSs was very low (less than 50 mg·L^−^^1^) and a probable deterioration of the membrane train may have affected the low performance.

The removal efficiency of COD and BOD were almost the same before and after the revamping of the plant. Although the overall removal rate of COD is similar, the difference between the two analyzed periods is shown in Figure 4. As of 2021, the biologically oxidized fraction of input COD (greater than 56%) appears to be higher than in the previous three-year period (less than 47%). At the same time, the fraction of input COD removed via sludge has decreased over time, from 37% in 2015 to 16% in 2023. Finally, the remaining portion of COD (21–28%) is released through the effluent as soluble non-biodegradable COD and has not changed over time. A possible explanation is to be found in the different sludge productions between the two periods. In fact, after the revamping of the plant, there was a decrease in the amount of sludge extracted (see Table 2), which resulted in a reduction in COD removed through sludge extraction. Furthermore, it is necessary to consider that, after the revamping, other categories of liquid waste (EWC code 16.10.02, aqueous liquid wastes) began to be treated, in addition to landfill leachate (EWC code 19.07.03). This may have affected the composition of COD entering the MBR plant and therefore the mass balance.

Regarding nitrogen, TN removal is variable over time, with similar results in the two considered periods. This means that the partial nitrification–denitrification process did not affect the performance of the plant despite the reduction in energy demand. The ammonia removal rate is around 98–99% in both periods, a result in accordance with [6], who used a pilot-scale nitritation–denitritation process to treat landfill leachate.

Finally, the TP removal efficiency has increased over the years, reaching an average value of almost 71% in the period of 2021–2022–2023, which is almost 20% more than in the previous three years. The analyzed biological reactor was been designed to remove TP; however, the intermittent aeration mode and the introduction of a liquid waste storage phase upstream of the inlet to the biological tank may have encouraged the growth of phosphorus-accumulating organisms (PAOs), improving phosphorus removal. During storage, in fact, conditions may be created for the fermentation of rapidly biodegradable COD to take place, resulting in the formation of VFA. In addition, the intermittent aeration mode may provide a good environment for the development of PAOs, which release/absorb P-PO_4_^3−^ during the anoxic/aerobic phases, respectively [12,13].

### 4.2. Membrane Separation

Permeate flux increased linearly from 2015 to 2023. This trend is attributable to the increase in the treated leachate flow rate (Figure 5) because of the directly proportional relationship between the two terms. The different behaviors between winter and summer are still connected to the inlet flow rate, characterized by a seasonal trend. In fact, in Italy, rainfall is more frequent during the winter than in the summer, so the leachate volume is usually higher.

Specific flux (or permeability) is also characterized by an increasing trend. Membrane permeability correlates with membrane fouling [14]. Among all factors, extracellular polymeric substances (EPS) have been recognized as the main components of fouling in MBRs. Fouling is more favored at low SRT, when the amount of EPS is greater, while at high SRTs, the concentration of EPS decreases as the biomass remains in the system for longer [15]. The plant analyzed in this work has a high SRT, which remained more stable and less variable after revamping. Furthermore, the presence of a storage tank allows for the biological section to be fed evenly, maintaining the same concentration of MLSSs as before the revamping. All of this may have led to less fouling of the membranes. As a result, no deterioration of permeability occurred. Considering the SRT of the system, it may be possible to work with lower MLSSs and evaluate the effect of this reduction on ultrafiltration performance.

### 4.3. Energy Consumption

The results showed that the introduction of alternated aeration in the biological compartment and the introduction of the partial nitrification process brought positive effects in terms of performances and energy/cost savings.

Regarding the MBR compartment, the introduction of the alternated aeration before the ultrafiltration unit lead to an improvement in hydraulic permeability, thus reducing the energetic cost associated with the lower TMP. This could be ascribed to a lower fouling rate due to a low EPS production, as previously suggested [16]. The change in aeration system after the revamping (i.e., from superficial aeration to submerged fine bubbles) resulted in an important improvement in dissolved oxygen transfer, thus leading to a further energy saving.

Regarding the reduction in the production of excess sludge reported in Table 2, it can be asserted that the introduction of the alternating cycles implied a decrease in the observed yield (Y_obs_) of microorganisms. This reduction can be attributed to the lower biomass produced under anoxic conditions compared to full aerobic conditions, as observed by other authors in the literature [17]. The reason for this occurrence could be that the value for the heterotroph anoxic yield was reduced compared to the aerobic yield as reported by [18], who found a value changing from 0.67 mg COD/mg COD to 0.53 mg COD/mg COD. This determines positive effects on the net sludge production, since, in most conventional nitrogen-removal activated sludge systems, the mass of sludge produced under anoxic conditions is lower compared to the one produced under aerobic conditions [19].

## 5. Conclusions

This paper described a case study involving a revamping of a full-scale membrane biological plant treating landfill leachate and other aqueous waste.

We observed that the biologically oxidized fraction of COD input appears to be higher after the revamping (56%) than before (47%). At the same time, removal of TN had similar results in the two considered periods, meaning that the partial nitrification–denitrification process did not affect the performance of the plant. In regard to the removal efficiency of TP, this surprisingly increased from about 52% to 71%. The main results are about the permeability and the energy consumption. Specific flux (or permeability) was characterized by an increasing trend, probably due to less fouling of the membranes. At the same time, we observed a decrease of 41.2%in the average specific consumption.

All the modifications led to a more stable process, less membrane fouling, and important energy savings.

## Figures and Tables

**Table 1:** Characteristics of the influent mixture of landfill leachates (data from 2015 to 2017 and from 2021 to 2023).

	Before Revamping			After Revamping		
Parameter	Mean Value ± SD	Minimum	Maximum	Mean Value ± SD	Minimum	Maximum
pH	8.1 ± 0.3	7.02	8.76	8.2 ± 0.21	7.45	8.87
TSSs (mg·L^−1^)	421 ± 808	55	7592	657 ± 629	142	6561
COD (mg·L^−1^)	9676 ± 4544	1649	34,767	6350 ± 2184	2676	27,233
BOD_5_ (mg·L^−1^)	3160 ± 1353	698	6333	2212 ± 1090	725	5920
TN (mg·L^−1^)	1826 ± 437	621	3205	1510 ± 463	656	5693
N-NH_4_^+^ (mg·L^−1^)	1404 ± 385	206	2593	1197 ± 373	186	4412
N-NO_3_^−^ (mg·L^−1^)	13.5 ± 12.7	0.05	95	20 ± 20	0.05	233
N-NO_2_^−^ (mg·L^−1^)	1.7 ± 2.3	0.05	25	0.17 ± 1.75	0.05	29.7
TP (mg·L^−1^)	47.1 ± 28.6	12	193	42 ± 26	7.68	195

**Table 2:** Main operating parameters of the oxidation/nitrification tank (data from 2015 to 2017 and from 2021 to 2023).

	Before Revamping			After Revamping		
Parameter	Mean Value	Minimum	Maximum	Mean Value	Minimum	Maximum
MLSSs (g·L^−1^)	23.8	16.6	30.5	24.0	18.3	31.2
Waste flow rate (TSSs/year)	415	250	528	188	126	240
SRT (d)	311	57	1583	254	142	548

## Data Availability

The original contributions presented in the study are included in the article, further inquiries can be directed to the corresponding author.
